# Clinical, contextual and hospital-level factors associated with escalation and de-escalation of empiric Gram-negative antibiotics among US inpatients

**DOI:** 10.1093/jacamr/dlad054

**Published:** 2023-05-13

**Authors:** Jonathan D Baghdadi, Katherine E Goodman, Laurence S Magder, Emily L Heil, Kimberly Claeys, Jacqueline Bork, Anthony D Harris

**Affiliations:** Department of Epidemiology and Public Health, University of Maryland School of Medicine, Baltimore, MD, USA; Department of Epidemiology and Public Health, University of Maryland School of Medicine, Baltimore, MD, USA; Department of Epidemiology and Public Health, University of Maryland School of Medicine, Baltimore, MD, USA; Department of Pharmacy Practice and Science, University of Maryland School of Pharmacy, Baltimore, MD, USA; Department of Pharmacy Practice and Science, University of Maryland School of Pharmacy, Baltimore, MD, USA; Department of Medicine, University of Maryland School of Medicine, Baltimore, MD, USA; Department of Epidemiology and Public Health, University of Maryland School of Medicine, Baltimore, MD, USA

## Abstract

**Background:**

Empiric Gram-negative antibiotics are frequently changed in response to new information. To inform antibiotic stewardship, we sought to identify predictors of antibiotic changes using information knowable before microbiological test results.

**Methods:**

We performed a retrospective cohort study. Survival-time models were used to evaluate clinical factors associated with antibiotic escalation and de-escalation (defined as an increase or decrease, respectively, in the spectrum or number of Gram-negative antibiotics within 5 days of initiation). Spectrum was categorized as narrow, broad, extended or protected. Tjur’s D statistic was used to estimate the discriminatory power of groups of variables.

**Results:**

In 2019, 2 751 969 patients received empiric Gram-negative antibiotics at 920 study hospitals. Antibiotic escalation occurred in 6.5%, and 49.2% underwent de-escalation; 8.8% were changed to an equivalent regimen. Escalation was more likely when empiric antibiotics were narrow-spectrum (HR 19.0 relative to protected; 95% CI: 17.9–20.1), broad-spectrum (HR 10.3; 95% CI: 9.78–10.9) or extended-spectrum (HR 3.49; 95% CI: 3.30–3.69). Patients with sepsis present on admission (HR 1.94; 95% CI: 1.91–1.96) and urinary tract infection present on admission (HR 1.36; 95% CI: 1.35–1.38) were more likely to undergo antibiotic escalation than patients without these syndromes. De-escalation was more likely with combination therapy (HR 2.62 per additional agent; 95% CI: 2.61–2.63) or narrow-spectrum empiric antibiotics (HR 1.67 relative to protected; 95% CI: 1.65–1.69). Choice of empiric regimen accounted for 51% and 74% of the explained variation in antibiotic escalation and de-escalation, respectively.

**Conclusions:**

Empiric Gram-negative antibiotics are frequently de-escalated early in hospitalization, whereas escalation is infrequent. Changes are primarily driven by choice of empiric therapy and presence of infectious syndromes.

## Introduction

Most patients with suspected bacterial infection are started on empiric antibiotic therapy before results from microbiological testing become available. Theoretically, empiric antibiotics should represent an educated guess at appropriate coverage based on the patient’s condition, medical history, prior culture results and the facility’s antibiogram.^1–3^ However, in administrative data, prescribing patterns vary widely between hospitals and are often unexplainable by patient-level factors.^1^

As microbiological test results become available and the patient’s clinical course evolves, it is standard practice to reassess and adjust empiric antibiotics.^4–8^ Antibiotic stewardship programmes can support this reassessment to escalate, de-escalate or otherwise improve antibiotic therapy as needed.^9,10^ Because audit and feedback is labour intensive, antibiotic stewardship programmes employ rules and heuristics to guide which cases will be reviewed.^11^ Examples may include auditing all patients for whom IV antibiotics are prescribed, all patients receiving a fluoroquinolone, or all patients with a bug-drug mismatch.^12^

In this study, we sought to facilitate the work of antibiotic stewardship programmes by identifying clinical factors that might predict when antibiotics require adjustment. Because it can be challenging to identify the appropriateness of antibiotic selection in retrospect, we examined instances in which empiric Gram-negative antibiotics were escalated or de-escalated shortly after initiation. To isolate clinical factors that might be ascertainable before microbiological test results become available, we focused on patient- and encounter-level characteristics from the first 2 days of hospitalization. The results of this analysis are intended to guide development, use, and implementation of antibiotic stewardship interventions to improve use of Gram-negative antibiotics in the hospital.

## Materials and methods

We performed a retrospective cohort study of patient discharge data from 920 hospitals contributing to the Premier Healthcare Database (‘the Premier database’), an all-payer deidentified administrative dataset containing approximately 25% of US inpatient admissions.^13^ The Premier database has been used frequently to evaluate patterns of antibiotic use across regions and hospitals.^14–21^ Hospitals contributing to the Premier database are diverse in geography, bed size and patient mix. This study did not include protected health information and was exempt from institutional review board review.

The study sample included all adult patients who were discharged from a Premier database hospital in calendar year 2019 and who were started on Gram-negative antibiotics during the ‘empiric window’, meaning within Days 0–2 of hospitalization.^1^ Hospital Day 0 was the day of presentation to or arrival at the hospital including time spent in the emergency department. Patients were excluded if discharged on the same day that empiric Gram-negative antibiotics were initiated.

**Figure 1.**
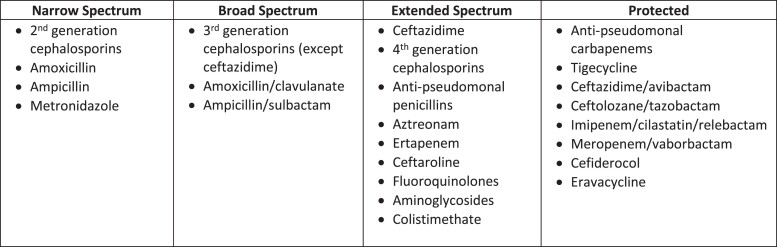
Categories of Gram-negative antibiotic spectrum of activity. Adapted from Moehring *et al.*^23^

The primary outcome was antibiotic escalation, defined as an increase in the overall spectrum of Gram-negative antibiotic therapy or an increase in the number of Gram-negative antibiotics, within 5 days of antibiotic initiation.^22,23^ Overall spectrum of activity was ranked using four categories (narrow, broad, extended, protected) and was determined by the broadest-spectrum Gram-negative antibiotic administered in a single day (see Figure 1 for categories).^1,23^ The secondary outcome was antibiotic de-escalation, defined as a decrease in the spectrum of activity or number of antibiotics, within 5 days of initiation.^23^ Definitions were operationalized in consultation with physician and pharmacist members of the antibiotic stewardship programme (J.D.B., J.B., E.L.H., K.C.) and literature review (see Appendix S1, available as Supplementary data at *JAC-AMR* Online).^22,23^ For example, a patient receiving combination therapy with the broadest category of empiric antibiotics (protected) could still undergo antibiotic escalation if an additional Gram-negative antibiotic were added. A patient receiving narrow-spectrum empiric monotherapy could undergo de-escalation if antibiotics were discontinued altogether. Additional examples of de-escalation, escalation, and changes that do not count as either are provided in Table 1.

**Table 1.** Examples of antibiotic escalation, de-escalation and changes that are neither^a^

Empiric Gram-negative-targeted antibiotics	Gram-negative-targeted antibiotics on third day of therapy	Classification
Ceftriaxone	Cefepime	Escalation
Cefepime	Ceftriaxone	De-escalation
Ceftriaxone	Amoxicillin/clavulanate	Neutral change
Ceftriaxone	Ceftriaxone + metronidazole	Escalation
Cefepime + metronidazole	Cefepime + levofloxacin	Neutral change
Piperacillin/tazobactam	Ceftriaxone + metronidazole	De-escalation
Piperacillin/tazobactam + amikacin	Ceftazidime/avibactam	Escalation

a Antibiotic changes were examined within 5 days of initiation.

In the primary analysis, parametric regression survival-time models using a Weibull distribution were used to estimate HRs for the association between predictor variables and outcomes. The first day of antibiotic therapy, defined as Antibiotic Day 1, was used as the time of study entry. Thus, Hospital Day 0 may count as Antibiotic Day 1 if antibiotics were started in the emergency department. Observations were censored at the time of first escalation, de-escalation or neutral change, after five consecutive days of therapy without a change, or at hospital discharge. In the case of early hospital discharge, censoring was assumed to be non-informative, and patients who were discharged within 2–5 days of antibiotic initiation were nonetheless able to contribute days ‘at risk’ for use within the model. A follow-up period of 5 days was selected to capture antibiotic changes early in therapy or after an abbreviated course for the presenting syndrome.^23^ Antibiotic changes after 5 days may represent completion of treatment or treatment of a different infectious process. Models were constructed using shared frailty to account for clustering within each hospital.

In a secondary analysis, logistic regression models with binary outcomes representing antibiotic escalation or de-escalation within 5 days of initiation were used to estimate the relative importance of variables by category, including patient characteristics, infection characteristics, hospital characteristics and characteristics of the empiric antibiotic regimen.^24^ Relative importance of each category was represented by discriminatory power as measured using the coefficient of discrimination, D, of a model including only variables from that category as covariates.^25,26^ The D statistic is calculated as the difference in average predicted probabilities between observations with and without the outcome of interest. For instance, if the model including patient characteristics predicted an average probability of escalation of 70% among patients whose antibiotics were escalated and a probability of 40% among patients whose antibiotics were not escalated, the D statistic would be 0.70–0.40 = 0.30. The proportion of discrimination explained by each category of variables was estimated by dividing the D statistic for the model including only that category of variables by the D statistic for the model including all categories of variables. So, if the D statistic for patient characteristics was 0.30 and the D statistic for the full model was 0.60, patient characteristics would be estimated to provide a proportion of discrimination explained of 0.30/0.60 = 50%.^24^ The 95% CIs for proportion of discrimination explained were bootstrapped with 100 repetitions. The proportion of discrimination explained can be summarized as the extent to which a group of variables predict whether a patient will have the outcome. Because it is known that antibiotic prescribing varies substantially among hospitals,^1^ the full model including all covariates and the model only including hospital characteristics included a random intercept for hospital.

Covariates were selected for inclusion in multivariable modeling based on prior work studying variation in selection of empiric Gram-negative antibiotics, with an emphasis on data that would be available within the first 2 days of hospitalization.^1^ Covariates included patient demographics, baseline health represented by number of comorbidities from the Elixhauser comorbidity index, history of previous readmission to the same hospital within 90 days, severity of illness represented by requirement for ICU admission or organ support in the first 2 days of hospitalization, infectious syndrome present on admission, hospital characteristics, and characteristics of the empiric antibiotic regimen.^27,28^ Diagnosis code sets representing urinary tract infection present on admission and pneumonia present on admission were from the Healthcare Utilization Project’s Clinical Classification Software Refined.^28^ Explicit diagnosis codes were used to represent sepsis, septic shock and bacteraemia, which have a high positive predictive value and tend to select for more severely ill patients.^29^ Because Premier does not contain microbiological data from most hospitals, covariates related to microbiological testing or hospital-level antibiograms were not included. All statistical tests were 2-tailed with a threshold of ≤0.05 for significance of *P* values. Analyses were performed using Stata/IC version 14.2 (StataCorp LLC, College Station, TX, USA).

### Data availability

Data and code sets will be made available upon request.

## Results

**Figure 2.**
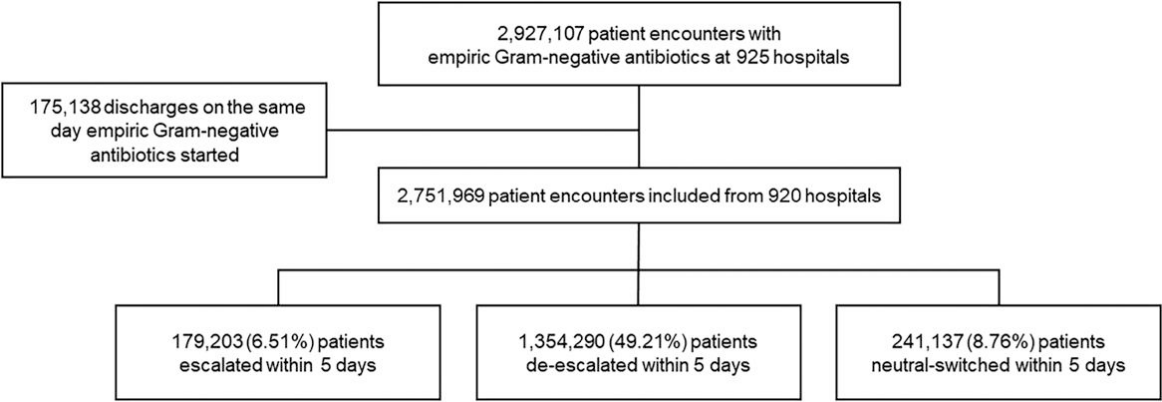
Cohort of hospitalized patients identified from the Premier Healthcare Database who received empiric Gram-negative antibiotics across 920 US hospitals in 2019.

Discharge data were included from 920 US hospitals. Hospitals were located in the US South (47.6%), Midwest (21.7%), Northeast (16.3%) and West (14.3%). Teaching facilities accounted for 45.0% of hospitals, and 30.6% were 500 beds or larger. We identified 2 751 969 patients who received empiric Gram-negative antibiotics during an inpatient admission in 2019 (Figure 2). The average age of patients was 63 years, and 54.7% were female. The median length of hospital stay was 4 days (IQR 3–7; 55.6% of patients were discharged before Hospital Day 5).

Of the initial regimens, 54.5% had an extended spectrum of activity (e.g. piperacillin/tazobactam), 34.6% were broad spectrum (e.g. ceftriaxone) and 7.4% were narrow spectrum (e.g. amoxicillin). A single Gram-negative antibiotic was included in 78.9% of the empiric antibiotic regimens, 18.3% included two, and 2.8% included three or more. The median total duration of Gram-negative antibiotics received was 4 days (IQR 2–6). Other characteristics of patients who received empiric Gram-negative antibiotics in this cohort have been reported previously.^1^

Patients who had their antibiotics changed within 5 days of initiation totalled 1 774 630 (64.5%), including 179 203 (6.51%) escalations, 1 354 290 (49.2%) de-escalations and 241 137 (8.76%) changes to a regimen with equivalent activity. The median time from initiation of empiric antibiotics to first antibiotic change was 2 days (IQR 2–4). Regarding escalations within 5 days, 71.1% involved an increase in the spectrum of antibiotic therapy, 28.9% involved an increase in the number of agents, and 12.6% involved both. As regards de-escalations within 5 days, 76.7% involved a decrease in the spectrum of antibiotic therapy, 23.3% involved a decrease in the number of agents, and 13.8% involved both. Characteristics of patients who had their antibiotics escalated or de-escalated within 5 days of initiation are provided in Table 2.

**Table 2.** Characteristics of hospitalized patients with escalation, de-escalation, equivalent change or no change of empiric Gram-negative-targeted antibiotics

	Antibiotics escalated (*n* = 179 203)	Antibiotics de-escalated (*n* = 1 354 290)	Antibiotics changed to equivalent (*n* = 241 137)	Antibiotics not changed (*n* = 977 339)
Age				
18–30 years old	11 037 (5.4%)	114 021 (55.5%)	15 122 (7.4%)	65 211 (31.8%)
31–40 years old	12 100 (5.7%)	112 943 (52.9%)	17 162 (8.0%)	71 345 (33.4%)
41–50 years old	16 100 (6.4%)	119 328 (47.6%)	22 171 (8.8%)	93 073 (37.1%)
51–60 years old	27 859 (6.5%)	207 789 (48.1%)	39 303 (9.1%)	157 284 (36.4%)
61–70 years old	37 686 (6.6%)	278 982 (48.7%)	52 037 (9.1%)	204 521 (35.7%)
71–80 years old	38 338 (6.8%)	273 040 (48.5%)	50 747 (9.0%)	201 104 (35.7%)
>80 years old	36 083 (7.0%)	248 187 (48.3%)	44 595 (8.7%)	184 801 (36.0%)
Gender				
Male	82 722 (6.6%)	592 057 (47.5%)	118 880 (9.5%)	453 593 (36.4%)
Female	96 481 (6.4%)	762 233 (50.7%)	122 257 (8.1%)	523 746 (34.8%)
Race				
Asian	3940 (7.4%)	27 089 (50.6%)	4893 (9.1%)	17 655 (33.0%)
Black	23 235 (6.4%)	181 503 (50.2%)	33 796 (9.3%)	122 990 (34.0%)
White	133 148 (6.5%)	1 002 473 (48.9%)	176 321 (8.6%)	737 315 (36.0%)
Other	18 880 (6.6%)	143 225 (49.8%)	26 127 (9.1%)	99 379 (34.6%)
Hispanic ethnicity	17 475 (7.1%)	120 176 (48.6%)	21 964 (8.9%)	87 521 (35.4%)
Public insurance	135 475 (6.7%)	987 279 (48.8%)	182 582 (9.0%)	717 163 (35.5%)
Point of origin				
Home	147 140 (6.6%)	1 098 103 (49.4%)	194 217 (8.7%)	782 487 (35.2%)
Long-term care	5147 (8.0%)	31 773 (49.5%)	7172 (11.2%)	20 091 (31.3%)
Hospital transfer	10 664 (6.1%)	71 676 (41.2%)	17 497 (10.1%)	74 032 (42.6%)
Infection present on admission				
Pneumonia	42 215 (7.8%)	227 278 (41.9%)	58 059 (10.7%)	215 272 (39.7%)
Urinary tract infection	63 093 (9.9%)	263 693 (41.6%)	70 163 (11.1%)	237 575 (37.4%)
Sepsis^a^	64 189 (9.8%)	292 172 (44.5%)	96 383 (14.7%)	204 441 (31.1%)
Bacteraemia^b^	3772 (9.4%)	19 258 (47.8%)	5858 (14.5%)	11 384 (28.3%)
Spectrum of empiric Gram-negative-targeted antibiotics				
Narrow	15 575 (7.6%)	132 977 (65.2%)	11 791 (5.8%)	43 624 (21.4%)
Broad	106 597 (11.2%)	348 908 (36.7%)	72 308 (7.6%)	423 401 (44.5%)
Extended	55 770 (3.7%)	810 940 (54.0%)	152 008 (10.1%)	482 326 (32.1%)
Protected	1261 (1.3%)	61 465 (64.2%)	5030 (5.3%)	27 988 (29.2%)
ICU utilization in the first 2 days of hospitalization				
Intensive care HD1, HD2	21 510 (7.2%)	151 818 (50.7%)	35 764 (12.0%)	90 090 (30.1%)
Intensive care HD1 only	5532 (4.9%)	62 323 (55.0%)	8591 (7.6%)	36 859 (32.5%)
Intensive care HD2 only	6314 (11.1%)	26 288 (46.2%)	9212 (16.2%))	15 035 (26.4%)
Any mechanical ventilation	11 260 (8.3%)	62 287 (46.0%)	18 426 (13.6%)	43 322 (32.0%)
Any vasopressors required	21 580 (6.1%)	208 237 (58.8%)	35 408 (10.0%)	89 062 (25.1%)

Values are reported as the frequency ‘*n*’ with the row percentage in parentheses. For variables with multiple levels, an overall chi-squared test was performed across all levels rather than a separate comparison at each level. HD1, Hospital Day 1; HD2, Hospital Day 2.

a Defined based on an explicit ICD-10 diagnosis code for septicaemia.

b Defined based on an ICD-10 diagnosis code of R78.81.

Among the 1 774 630 patients whose antibiotics were escalated, de-escalated or changed within 5 days of antibiotic initiation, 168 874 (9.5% of 1.77 M) were started on antibiotics on Hospital Day 0, 1 371 516 (77.3% of 1.77 M) were started on antibiotics on Hospital Day 1, and 234 240 (13.2% of 1.77 M) were started on antibiotics on Hospital Day 2. As regards antibiotic changes, including escalations, de-escalations and neutral changes, 65.1% occurred the day after antibiotics were initiated. An escalation, de-escalation, or equivalent change occurred for 65.5% of antibiotics started on Hospital Day 0, 67.6% of antibiotics started on Hospital Day 1, and 50.6% of antibiotics started on Hospital Day 2 (*P* < 0.001 by chi-square testing).

**Figure 3.**
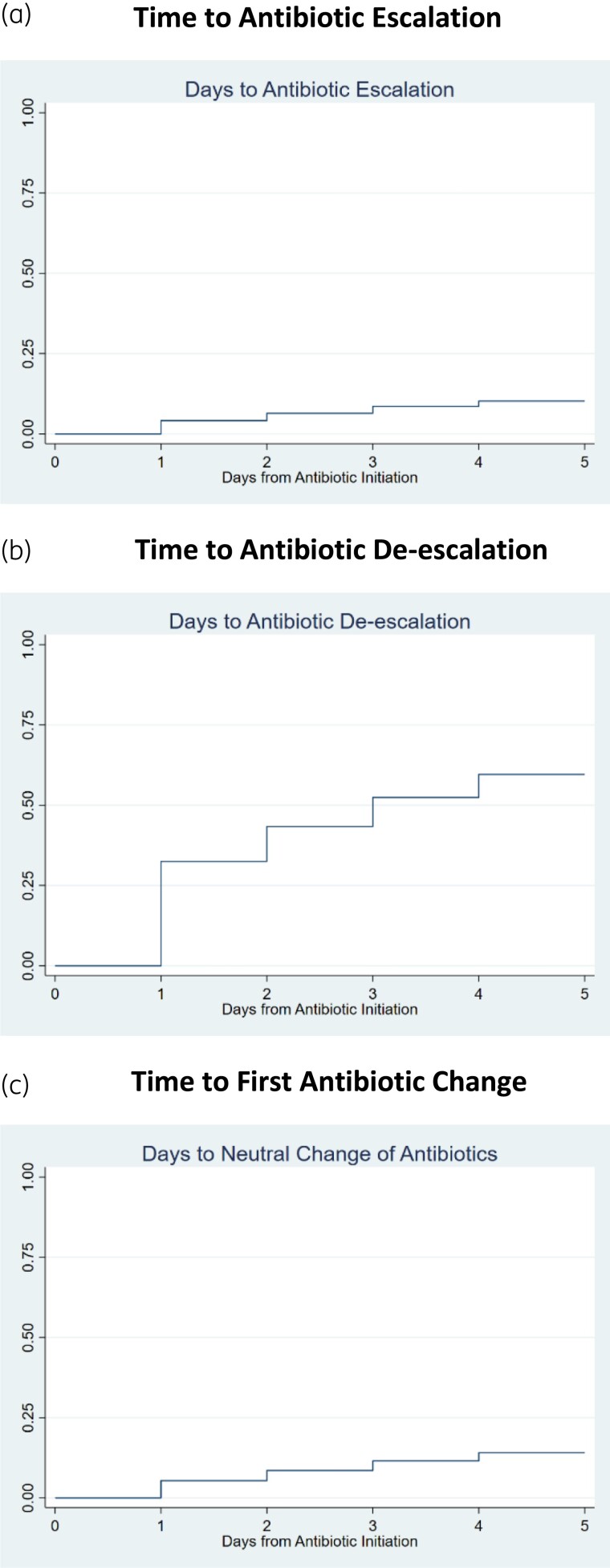
Kaplan–Meier curves representing time to antibiotic escalation (a), time to de-escalation (b), and time to neutral change to a regimen with equivalent activity (c). Observations were censored at the time of first escalation, de-escalation or neutral change, after five consecutive days of therapy without a change, or at hospital discharge.

Kaplan–Meier curves representing time to antibiotic escalation, de-escalation or first antibiotic change are provided in Figure 3. Factors associated with time to escalation are listed in Table 3. Escalation was more likely to occur when the initial regimen was narrow spectrum (HR 19.0; 95% CI: 17.9–20.1), broad spectrum (HR 10.3; 95% CI: 9.78–10.9) and extended spectrum (HR 3.49; 95% CI: 3.30–3.69), relative to patients whose initial therapy included a protected agent. Escalation was less likely to occur when initial antibiotics included combination therapy (HR 0.78 per each additional agent; 95% CI: 0.76–0.79) and when initial antibiotics were started after Hospital Day 0 (HR 0.68 if antibiotics were initiated on Hospital Day 1, 95% CI: 0.67–0.69; HR 0.36 if antibiotics were initiated on Hospital Day 2, 95% CI: 0.36–0.37). Escalation was more likely to occur at hospitals where empiric antibiotics were more frequently prescribed from the protected category (HR 21.0 per one percentage point increase in proportion of empiric antibiotics from the protected category; 95% CI: 11.5–38.3), extended-spectrum category (HR 6.11 per one percentage point increase; 95% CI: 4.38–8.52) or broad-spectrum category (HR 1.89 per one percentage point increase; 95% CI: 1.33–2.68). Patients were more likely to have their empiric Gram-negative antibiotics escalated when presenting with community-onset sepsis (HR 1.94; 95% CI: 1.91–1.96), with urinary tract infection (HR 1.36; 95% CI: 1.35–1.38), or when transferred into the ICU on Hospital Day 2 (HR 1.66; 95% CI: 1.61–1.70). Patients were less likely to have their empiric Gram-negative antibiotics escalated during an elective admission (HR 0.51; 95% CI: 0.49–0.52).

**Table 3.** Factors associated with escalation or de-escalation of empiric Gram-negative antibiotics in survival analysis

Factor	Antibiotic escalation (HR, 95% CI)	Antibiotic de-escalation (HR, 95% CI)
Age (per 10 year increase)	0.97 (0.97–0.97)	0.99 (0.99–0.99)
Female gender	0.91 (0.90–0.92)	1.11 (1.11–1.11)
Race/ethnicity		
Asian	1.10 (1.06–1.13)	1.03 (1.02–1.05)
Black	0.95 (0.94–0.97)	1.01 (1.01–1.02)
Hispanic	1.06 (1.04–1.08)	1.00 (1.00–1.01)
White non-Hispanic	reference	reference
Elixhauser comorbidity score	1.00 (1.00–1.01)	0.98 (0.98–0.98)
Admission type		
Urgent	0.91 (0.89–0.92)	0.91 (0.90–0.91)
Elective	0.51 (0.49–0.52)	1.66 (1.65–1.67)
Emergent	reference	reference
Pre-hospital origin		
Home	reference	reference
Other acute care hospital	0.99 (0.97–1.01)	0.66 (0.65–0.67)
Long-term care	1.19 (1.16–1.23)	1.00 (0.99–1.01)
Other source	1.02 (1.00–1.04)	0.89 (0.88–0.89)
Infectious syndrome present on admission		
Pneumonia	1.00 (0.98–1.01)	0.88 (0.87–0.88)
Urinary tract infection	1.36 (1.35–1.38)	0.98 (0.98–0.99)
Sepsis^a^	1.94 (1.91–1.96)	0.84 (0.83–0.84)
Bacteraemia^b^	1.62 (1.57–1.68)	0.98 (0.96–0.99)
Spectrum of empiric Gram-negative antibiotics		
Narrow	19.0 (17.9–20.1)	1.67 (1.65–1.69)
Broad	10.3 (9.78–10.9)	0.71 (0.70–0.72)
Extended	3.49 (3.30–3.69)	0.99 (0.98–1.00)
Protected	reference	reference
Number of Gram-negative antibiotics in empiric regimen	0.78 (0.76–0.79)	2.62 (2.61–2.63)
Day of empiric antibiotic initiation		
Hospital Day 0	reference	reference
Hospital Day 1	0.68 (0.67–0.69)	1.22 (1.21–1.23)
Hospital Day 2	0.36 (0.36–0.37)	1.18 (1.18–1.19)
Intensive care utilization in the first 2 days of hospitalization		
Admitted to intensive care HD1, HD2	0.95 (0.93–0.96)	0.94 (0.93–0.95)
Intensive care HD1 then transferred out	0.86 (0.84–0.88)	1.13 (1.12–1.14)
Transferred into intensive care HD2	1.66 (1.61–1.70)	0.88 (0.87–0.89)
Any mechanical ventilation	1.29 (1.26–1.32)	0.82 (0.82–0.83)
Any vasopressors required	1.22 (1.20–1.24)	1.15 (1.14–1.15)
Number of major surgeries in first two hospital days	0.98 (0.97–0.99)	1.03 (1.03–1.03)
Hospital characteristics		
Average case-mix index	0.91 (0.83–0.98)	1.21 (1.12–1.30)
Proportion of patients admitted by acute transfer	0.63 (0.51–0.79)	0.51 (0.43–0.61)
Proportion of patients on public insurance	1.00 (0.998–1.00)	0.997 (0.995–0.999)
Proportion of empiric antibiotics in broad category	1.89 (1.33–2.68)	0.71 (0.53–0.94)
Proportion of empiric antibiotics in extended category	6.11 (4.38–8.52)	0.77 (0.59–1.02)
Proportion of empiric antibiotics in protected category	21.0 (11.5–38.3)	0.85 (0.48–1.48)

HRs and 95% CIs were estimated using a multivariable parametric survival-time regression model. Other variables included in the multivariable model included month of admission and hospital characteristics: geographical division, bed size, teaching status, urban versus non-urban. Hospital Day 0 represents the day of presentation to or arrival at the hospital. HD1, hospital Day 1; HD2, hospital Day 2.

a Defined based on an explicit ICD-10 diagnosis code for septicaemia.

b Defined based on an ICD-10 diagnosis code of R78.81.

Factors associated with time to de-escalation are also listed in Table 3. Relative to initial therapy with a protected agent, narrow-spectrum initial antibiotics were more likely to be de-escalated (HR 1.67; 95% CI: 1.65–1.69) and broad-spectrum initial antibiotics were less likely to be de-escalated (HR 0.71; 95% CI: 0.70–0.72). Extended-spectrum initial antibiotics were roughly equivalent in likelihood of de-escalation as agents from the protected category (HR 0.99; 95% CI: 0.98–1.00). Initial combination therapy increased likelihood of de-escalation (HR 2.62 per each additional agent; 95% CI: 2.61–2.63). Other than characteristics of initial therapy, de-escalation was more likely to occur during an elective admission (HR 1.66; 95% CI: 1.65–1.67) and less likely to occur after hospital transfer (HR 0.66; 95% CI: 0.65–0.67). Patients admitted to hospitals with a higher proportion of their admissions via acute care transfer were also less likely to undergo de-escalation (HR 0.51 per one percentage point increase in proportion of admissions via acute care transfer; 95% CI: 0.43–0.61).

A comparison of the relative importance of variables is included in Table 4. In the full multivariable model including all covariates, the difference in average predicted probability of Gram-negative antibiotic escalation between patients who underwent escalation and those who did not was 5.6%. By comparison, the model including only covariates related to characteristics of the empiric antibiotic regimen produced a D statistic of 2.8%, which accounted for 51% of the discrimination explained by the full model. Models including only patient characteristics, infection characteristics and hospital characteristics produced D statistics of 0.35%, 1.2% and 0.32%, respectively. For antibiotic de-escalation, average predicted probabilities from the full multivariable model among patients who did and did not have the outcome differed by 17.4%. The model including only covariates related to characteristics of the empiric antibiotic regimen produced a D statistic of 12.8%, which accounted for 74% of the discrimination explained by the full model. Models including only patient characteristics, infection characteristics and hospital characteristics produced D statistics of 2.3%, 2.7% and 2.1%, respectively.

**Table 4.** Relative discriminatory power of variables for whether antibiotics are escalated or de-escalated

	Antibiotic escalation		Antibiotic de-escalation	
Covariates^a^	Tjur’s D statistic (95% CI)	Proportion of discrimination explained	Tjur’s D statistic (95% CI)	Proportion of discrimination explained
Patient-related	0.35% (0.34–0.36)	6.3%	2.3% (2.3–2.4)	13.4%
Infection-related	1.2% (1.1–1.2)	20.8%	2.7% (2.6–2.7)	15.2%
Empiric antibiotics	2.8% (2.8–2.8)	50.6%	12.8% (12.7–12.8)	73.5%
Hospital-related	0.32% (0.31–0.34)	5.8%	2.1% (2.0–2.1)	11.8%
Full model	5.6% (5.5–5.7)	100%	17.4% (17.3–17.5)	100%

Tjur’s D statistic represents the ability of a model to discriminate between patients who did and did not have the outcome of interest.

a *Patient characteristics*: Age, sex, race, public insurance, pre-hospital origin, admission type, number of Elixhauser comorbidities. *Infection characteristics*: Pneumonia (present on admission or hospital acquired), urinary tract infection (present on admission or hospital acquired), sepsis (present on admission or hospital acquired), bacteraemia (present on admission, or hospital acquired), number of surgeries required in the first 2 days of hospitalizations, requirement of mechanical ventilation in the first 2 days of hospitalization, requirement of vasopressor support in the first 2 days of hospitalization, transfer into or out of the ICU during the first 2 days of hospitalization. *Empiric antibiotics*: Number of agents included in initial antibiotic regimen, spectrum of activity of initial antibiotic regimen, hospital day on which empiric antibiotics were initiated. *Hospital characteristics*: Percentage of admissions via acute care transfer, average case-mix index, percentage of patients receiving public insurance, bed size, urban setting, teaching status, average spectrum of activity of initial antibiotic regimens.

## Discussion

When bacterial infection is suspected in a hospitalized patient, it is standard practice to start empiric antibiotics before the pathogen and site of infection are known. To our knowledge, this study is the largest to date that examines what happens next. For over 2.7 million patients admitted to 920 hospitals in a single year, about half of empiric antibiotics were de-escalated within 5 days. Escalation was much less frequent. These findings corroborate our clinical experience and the observations of others that clinicians would rather prescribe empiric antibiotics that are too broad than too narrow.^30^

Changes to empiric therapy were most frequent on the day after Gram-negative antibiotics were initiated. In some cases, initial antibiotic selection may have been suboptimal, and antibiotic changes were driven by new data or evolution of the patient’s condition. However, in many cases, we suspect that these early antibiotic changes were driven primarily by hand-offs between care teams or within the same care team.^31^ When several options are available, selection of empiric antibiotics may not be perfectly rational and instead can be influenced by the prescriber’s emotional state or personal preference.^32,33^ To support better decision-making, antibiotic selection should utilize the expertise of an interdisciplinary team.^34,35^ In the negotiation between the prescribing clinician and antibiotic stewardship team, the empiric antibiotic regimen may be considered analogous to a starting offer.^30^ Though multiple changes can be perceived as lacking clinical ‘finesse’, clinicians should not be expected to predict the perfect regimen upfront based on incomplete data. Revisiting and adjusting antibiotic selections as needed supports safe, high-quality care.

Antibiotic de-escalation is known to be influenced by both the empiric regimen and the clinical syndrome.^36,37^ By comparing D statistics, we were able to evaluate the relative importance of groups of variables in discriminating between patients who will or will not have their empiric antibiotics changed shortly after initiation. Based on this analysis, among patient, hospital and clinical factors that are knowable before microbiological test results become available, the primary determinants of antibiotic changes for both escalation and de-escalation were characteristics of the empiric regimen. From a stewardship perspective, this finding is reassuring in that it suggests suboptimal empiric therapy is frequently improved. At the same time, our results suggest potential opportunities to reduce the work of stewardship teams. For instance, a major driver of antibiotic de-escalation was use of combination empiric therapy. Implementation of protocols or policies that gently discourage clinicians from using multiple Gram-negative antibiotics in combination would likely pre-empt the need for streamlining later. Combination therapy is rarely indicated, except in the setting of pathogens with specific resistance patterns, such as carbapenem-resistant *Acinetobacter baumanii*.^7,8^

Studies examining antibiotic change behaviours tend to focus on de-escalation rather than escalation.^36–42^ A strength of this study is that we leveraged a large dataset and addressed this gap in the literature by exploring factors associated with antibiotic escalation. Results of this analysis were both predictable and surprising. For instance, it was predictable that the infectious syndromes associated with severe illness in our model—sepsis and bacteraemia—were associated with increased likelihood of antibiotic escalation. On the other hand, it was surprising that urinary tract infection present on admission was associated with antibiotic escalation, whereas pneumonia present on admission was not. Instead, patients presenting with pneumonia were more likely to have their antibiotics de-escalated. The discrepancy in antibiotic changing behaviours between patients with urinary tract infection and patients with pneumonia suggests the potential role that positive cultures play in driving antibiotic use. Even when symptoms are absent or not consistent with an infection, positive cultures are a strong stimulus to prescribe antibiotics.^43^ Whereas urinary tract infection is frequently diagnosed based on a positive culture, no pathogen is identified for a large proportion of pneumonias.^44^ Further, though urinary tract infections are commonly perceived as being low risk, resistance is increasingly recognized.^45–47^ To address antibiotic use for urinary tract infections, stewardship teams likely need to emphasize appropriate culturing practices, including only obtaining cultures from symptomatic patients.^48^

Though predictable, it is notable that empiric Gram-negative antibiotics were more likely to be escalated and less likely to be de-escalated among patients presenting with sepsis than among patients without sepsis present on admission. Increased likelihood of antibiotic escalation may be attributable to the inherent instability of septic patients and the diagnostic uncertainty that often accompanies their initial presentation.^49^ However, protocols for early sepsis care prioritize reflexively broad antibiotic therapy with the understanding that the plan of care can be re-evaluated later.^50^ Given that one-third of patients presenting with sepsis will later be found to have non-infectious conditions or non-bacterial infection,^51^ decreased likelihood of antibiotic de-escalation in this patient population suggests that this re-evaluation is not occurring consistently. Instead, our findings may represent evidence of diagnostic inertia.^52^ Until practical guidance on how and when to re-evaluate ongoing need for antibiotics is built into formal protocols for sepsis care, such as the Centers for Medicare and Medicaid Services SEP-1 bundle, hospitals may encounter antibiotic overuse in this patient population.

### Limitations

The major limitation of this study is that most hospitals do not contribute microbiological testing data to the Premier database. Though microbiological test results are a major driver of antibiotic changes, we specifically designed our study to examine how patient- and hospital-level factors that are knowable *before* results from microbiological testing predict antibiotic changes. Though we did not have access to hospital antibiograms, we used statistical techniques to account for clustering of data at the hospital level. Given the large size of the dataset, in this discussion we emphasized findings that may have clinical significance for antibiotic stewardship programmes, in addition to statistical significance.

Additionally, our study is subject to the limitations of administrative data without chart review. Not all patients diagnosed with infection may truly have bacterial infection, and present-on-admission coding may only be accurate in about 70% of cases.^53^ Specifically, we did not have access to data related to symptoms, and thus patients with asymptomatic bacteriuria may have been misclassified as having urinary tract infections. Some instances of antibiotic de-escalation may have reflected perioperative antibiotics, though we controlled for number of surgeries during the first 2 days of hospitalization in multivariable analysis. Further, over half of the patients in our sample were discharged before Hospital Day 5 and thus were non-informatively censored. Based on the limitations of our dataset, we were unable to detect medications administered outside of the hospital encounter, including antibiotics prescribed prior to admission or after discharge, changes to antibiotic dosing during hospitalization, or specific antibiotic administration times. Finally, it was beyond the scope of this study to examine outcomes of care. Thus, we cannot assess whether antibiotic choices were appropriate or inappropriate.

### Conclusions

De-escalation of empiric Gram-negative antibiotics is common among hospitalized patients. Antibiotic escalation and other antibiotic changes are much less common. Understanding predictors of escalation and de-escalation can help target stewardship efforts. For instance, stewardship programmes may consider re-examining protocols for combination therapy and empiric antibiotics for urinary tract infections. Combination therapy is more likely to be de-escalated than monotherapy, whereas empiric antibiotics targeting urinary tract infections are more likely to be escalated than antibiotics for other infectious syndromes.

## Supplementary Material

dlad054_Supplementary_Data

## Supplementary data

Appendix S1 is available as Supplementary data at *JAC-AMR* Online.
