# Supplementary material for: Clinical, contextual and hospital-level factors associated with escalation and de-escalation of empiric Gram-negative antibiotics among US inpatients
Source: JAC Antimicrob Resist. 2023 May 13;5(3):dlad054. doi: 10.1093/jacamr/dlad054 (PMC10182731; doi:10.1093/jacamr/dlad054)
Supplement: dlad054_Supplementary_Data [file dlad054_supplementary_data.docx]

**Appendix 1. Definitions of Escalation, De-escalation, and Neutral Changes (Switch)**

**AL** = numerical value representing the antibiotic level of broadest Gram-negative antibiotic use

- 4 = extremely broad
- 3 = extended
- 2 = narrower
- 1 = narrowest
- 0 = no Gram-negative antibiotics
- +Modifiers for multiple antibiotics
  - +0.1 = two antibiotics in combination
  - +0.2 = three antibiotics in combination
  - +0.3 = four antibiotics in combination
  - And so on…
- Examples:
  - Piperacillin/tazobactam = 3
  - Cefepime + metronidazole = 3.1
  - Ceftriaxone + metronidazole = 2.1
  - Cefepime + ciprofloxacin + metronidazole = 3.2

**d** = antibiotic day; the first day of empiric Gram-negative antibiotics is d=1

- Examples:
  - Empiric antibiotics are started on HD2. HD2 is counted as d=1.

**AL[d]** = antibiotic level on a given antibiotic day; the antibiotic level on d=1 is AL[1]

- Examples:
  - Empiric meropenem is started on HD2. AL[1] = 3.

**Sustained antibiotic change** = new antibiotic started on d=1 is continued on d=(x + 1)

- Examples:
  - d=1: meropenem; d=2: meropenem + ceftriaxone, d=3: ceftriaxone.
    - Initiation of ceftriaxone is a sustained change on d=2.
  - d=1: meropenem; d=2: meropenem + ceftriaxone; d=3: meropenem + ceftriaxone.
    - Initiation of ceftriaxone is a sustained change on d=2.
  - d=1: meropenem; d=2 meropenem + ceftriaxone; d=3 meropenem.
    - Initiation of ceftriaxone on d=2 is not a sustained change.
- An exception is created for aminoglycosides, which are often dosed at extended intervals. A single dose of an aminoglycoside is considered a sustained changed.
  - d=1: meropenem; d=2 meropenem + gentamicin; d=3 meropenem.
    - Initiation of gentamicin on d=2 is a sustained change.

**Discharge antibiotic** = new antibiotic started on d = max(service day)

- Examples:
  - d=1: meropenem; d=2: meropenem + ceftriaxone; d=3: no longer hospitalized
    - Ceftriaxone is a discharge medication.

**DL** = numerical value representing the antibiotic level of discharge antibiotics

- Scored using same values as AL, but only discharge antibiotics are counted.

Criteria for escalation can be met two ways:

**Escalation 1** = {sustained antibiotic change on d=x and (AL[x + 1]) > (AL[x – 1])}

- Examples:
  - d=1: meropenem; d=2: meropenem + ceftriaxone; d=3: meropenem + ceftriaxone
    - Escalation attributed to d=2
  - d=1: meropenem; d=2: meropenem/vaborbactam; d=3: meropenem/vaborbactam
    - Escalation attributed to d=2

**Escalation 2** = {aminoglycoside administered on d=x and (AL[x]) > (AL[x – 1])}

- Examples:
  - d=1: meropenem; d=2: meropenem + gentamicin
    - Escalation attributed to d=2

**Escalation 3** = DL[x] > AL[x – 1]

- Examples:
  - d=1: meropenem; d=2: meropenem + ciprofloxacin + metronidazole; d=3: no longer hospitalized
    - Ciprofloxacin and metronidazole are discharge antibiotics. When a switch occurs on the last day of hospitalization, only new antibiotics started on that day are used to determine the antibiotic level. In this case, DL[x] = 3.1, while AL[x-1] = 3. Escalation is attributed to d=2.

Criteria for de-escalation can be met two ways:

**De-escalation 1** = AL[x]) < (AL[x – 1]

- Examples:
  - d=1: meropenem; d=2: ceftriaxone
    - De-escalation attributed to d=2
  - d=1: meropenem + gentamicin; d=2: meropenem
    - De-escalation attributed to d=2

**De-escalation 2** = {sustained antibiotic change on d=x and (AL[x + 1]) < (AL[x – 1])}

- Examples:
  - d=1: meropenem; d=2: meropenem + ceftriaxone; d=3: ceftriaxone
    - De-escalation attributed to d=2

**De-escalation 3** = DL[x] < AL[x – 1]

- Examples:
  - d=1: meropenem; d=2: meropenem + ceftriaxone + metronidazole; d=3: no longer hospitalized
    - Ceftriaxone is a discharge antibiotic. When a switch occurs on the last day of hospitalization, only new antibiotics started on that day are used to determine the antibiotic level. In this case, DL[x] = 2.1 and AL[x-1] = 3. De-escalation attributed to d=2.

Criteria for switch and directionality of switch

**Switch** = antibiotics on d=x ≠ antibiotics on d=(x – 1)

**Equivalent switch 1 =** antibiotics are switched and AL[x] = AL[x-1]

- Examples:
  - d=1: meropenem; d=2: meropenem + ceftriaxone; d=3: meropenem
    - Addition of ceftriaxone was not a sustained change. Antibiotics were switched on d=2, but not escalated.

**Equivalent switch 2 =** antibiotics are switched and DL[x] = AL[x-1]

- Examples:
  - d=1: meropenem; d=2: meropenem + cefepime; d=3: no longer hospitalized
    - Cefepime is a discharge antibiotic. DL[x] = 3 and AL[x-1] = 3.

**Escalation 3** = {discharge antibiotice on d=x and (AL[x + 1]) > (AL[x – 1])}

- Examples:
  - d=1: meropenem; d=2: meropenem + ceftriaxone; d=3: meropenem + ceftriaxone
    - Escalation attributed to d=2

**Escalatory/positive switch** = Escalation 1-3

**De-escalatory/negative switch** = De-escalation 1-3
